# Coated Urea Materials for Improving Yields, Profitability, and Nutrient Use Efficiencies of Aromatic Rice

**DOI:** 10.1002/gch2.201900013

**Published:** 2019-09-05

**Authors:** Yashbir Singh Shivay, Vijay Pooniya, Madan Pal, Prakash Chand Ghasal, Ramswaroop Bana, Shankar Lal Jat

**Affiliations:** ^1^ Division of Agronomy ICAR–Indian Agricultural Research Institute New Delhi 110012 India; ^2^ ICAR–Indian Institute of Farming Systems Research Modipuram Meerut 250110 India; ^3^ ICAR–Indian Institute of Maize Research Ludhiana Punjab 141004 India

**Keywords:** aromatic rice, boron‐coated urea, sulfur‐coated urea, zinc‐coated urea

## Abstract

Intensive cultivation and introduction of input‐responsive high‐yielding varieties with application of major nutrients in rice–wheat rotation of Indo‐Gangetic plains (IGPs) lead to multiple nutrient deficiencies. A survey of Indian soils has shown that 40% are deficient in available zinc (Zn), 33% in sulfur (S), and 33% in boron (B). Studies have indicated that application of these nutrients with major nutrients can improve the crop productivity. Keeping the importance of aromatic rice in view, coated‐urea materials and their effects on rice yields, nitrogen (N), and Zn content in different parts and input economics are evaluated. Three field trials are conducted on aromatic rice to test boron‐coated urea (BCU), sulfur‐coated urea (SCU), and zinc‐coated urea (ZnCU) in 2013 and 2014. Results indicate that the highest yields are obtained with 0.5% BCU, 5.0% SCU, and 2.5% ZnCU as zinc sulfate heptahydrate. These treatments increase grain yield by 13%, 25%, and 17.9% over prilled urea (PU). Moreover, 0.5% BCU, 5% SCU, and 2.5% ZnCU register the highest N, S, and Zn contents in bran, husk, grain, and straw. Coated‐urea materials also improve use efficiencies and harvest index of N and Zn over PU. The findings of this study suggest that 0.5% boron, 5.0% sulfur, or 2.5% zinc‐coated urea show improvement in returns and benefit–cost ratio in aromatic rice of western IGPs.

## Graphical abstract

**Micro–(Zn, B) and secondary (S) nutrients coating onto prilled urea** leads to maximized benefits and increased N/Zn–use efficiencies.

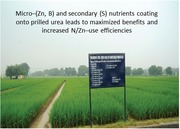

## 1 Introduction

Rice (*Oryza sativa* L.) is the most important food crop not only in Asia but also in the entire world as it feeds almost half of the world population on a daily basis.^1^ It satiates the hunger of nearly 60% Indian population and accounts for ≈40% of the total food grain production of the country.^2^ Rice is being cultivated under diverse agroecologies varying from irrigated, upland, rainfed lowland to flood‐prone rice ecosystems. To overcome the production vulnerabilities in rice, the scientific taskforce at the Indian Council of Agricultural Research has developed many high yielding input responsive cultivars, having productivity more than 6.0 t ha^−1^ besides good cooking quality characteristics. Despite significant progress, the average productivity of rice in India is low. One of the prime reasons for lower productivity of Indian rice is improper nutrient management. Farmers are predominantly applying major nutrients especially nitrogen and phosphorous without considering the importance of micronutrients (B, Zn) and other secondary (S) nutrients.^3^ In a survey of Indian soils encompassing 1.7 lakh soil samples, about 40% of the samples were deficient in available Zn, and the severity was least in Himachal Pradesh (1.4%) and highest in Tamil Nadu (65.5%).^4^ Cereals cultivated on Zn‐deficient soils have low Zn content and consequently bioavailability. Zn inadequacy accounts for about 4% of global morbidity and mortality among children under five years of age.^5^ Application of Zn fertilizers in rice crop significantly improves the grain yield and grain Zn concentration.^1^, ^5^ In India, zinc sulfate and zinc oxide are two most commonly available Zn fertilizers. It is also reported that application of Zn impregnated urea improved the aromatic rice grain yield by 29% compared to prilled urea (PU)^6^ in addition to agronomic efficiency of applied Zn and N^7^, ^8^ and Zn concentration in grain.^9^ Besides, coated urea requires less amount of Zn to fulfill crop demand.^10^, ^11^

On an average, ≈33% of Indian soils are deficient in S and it is widespread in coarse textured alluvial, red and lateritic, leached acidic and hill soils, and black clayey soils.^12^ What's more, the deficiency of S is emerging fast in areas where S‐free fertilizers like DAP, urea, etc., are being used continuously. The coating of urea with S is a possible solution to reduce N loss and improve use efficiency.^13^ Application of S‐coated urea increased rice dry matter yields by 55–68%^13^, ^14^ and doubled N recovery over PU.^15^ However, very limited literature is available on effect of application of graded dose of SCU on grain yield, nutrient use efficiency, and input economics in the rice crop.

In India, ≈33% of soil samples collected from different locations were deficient in B.^16^ Boron‐deficient soils include those which are inherently low in B, calcareous and coarse textured soils, and those high in clay content. Therefore, application of B in these soils significantly improves plant growth, yield traits, and yield of crops. Soil application of B improved crop growth and grain yield in maize.^17^, ^18^ Similarly, application of BCU significantly increased grain yield and N recovery efficiency in spring wheat.^19^ The field experiments on BCU, SCU, and ZnCU were set up with the aim to study the response of aromatic rice to varying Zn, B, and S‐coated urea levels besides estimating the use efficiencies of applied coated urea/fertilizers and economic evaluation of different coated fertilizer materials.

## 2 Results

### 2.1 Rice Yield and Yield Attributes

Application of prilled urea (PU) significantly increased the leaf area index (LAI) at 65 d DAT as compared to absolute control. Similarly, BCU at 0.5% (1.40 kg B ha^−1^), 0.4% (1.12 kg B ha^−1^), and 0.3% (0.84 kg B ha^−1^) increased the LAI (**Table** **1**). Application of SCU produced significantly higher LAI over PU and absolute control, and the highest LAI was recorded at 5.0% level (15.7 kg S ha^−1^). Urea coating with 2.5% Zn (zinc oxide (ZnO)) resulted in highest LAI and was similar to other treatments except PU and absolute control. Application of 0.5% BCU produced longest panicle being at par with BCU materials but significantly longer than PU and absolute control. Different SCU and PU treatments recorded similar panicle length and the longest was achieved with 5.0% SCU. In case of ZnCU, the highest panicle length was observed in 2.5% ZnCU (zinc sulfate heptahydrate (ZnSHH)) and was identical to other concentrations except 0.5% ZnCU (ZnO), PU, and absolute control.

**Table 1:** Effect of boron‐coated urea, sulfur‐coated urea, and Zn‐coated urea materials on yield parameters and yields in aromatic rice. Means in a column with at least one letter common are not statistically significant using Fisher's least significant difference

Treatment	Amount of B/S/Zn added [kg ha^−1^]	LAI at 65 DAT	Panicle length [cm]	Grain weight/panicle [g]	1000 − grain weight [g]	Grain yield [t ha^−1^]	Straw yield [t ha^−1^]
Absolute control	–	1.70^E^	24.2^C^	0.67^D^	18.5^B^	2.15^E^	6.53^E^
Prilled urea	–	2.21^D^	26.5^B^	0.75^C^	20.4^AB^	3.08^D^	9.12^D^
0.1% BCU	0.28	2.53^C^	27.3^AB^	0.78^C^	20.9^A^	3.19 (3.57)^a)^ ^CD^	9.42 (3.29)^CD^
0.2% BCU	0.56	2.65^B^	27.5^AB^	0.85^B^	21.0^A^	3.25 (5.52)^BCD^	9.68 (6.14)^BCD^
0.3% BCU	0.84	2.68^AB^	27.7^AB^	0.88^AB^	21.2^A^	3.34 (8.44)^ABC^	9.96 (9.21)^ABC^
0.4% BCU	1.12	2.72^AB^	27.9^AB^	0.90^A^	21.3^A^	3.43 (11.4)^AB^	10.25 (12.4)^AB^
0.5% BCU	1.40	2.75^A^	28.3^A^	0.91^A^	21.5^A^	3.48 (13.0)^A^	10.36 (13.6)^A^
Absolute control	–	1.70^F^	24.2^B^	0.67^E^	18.5^B^	2.15^D^	6.53^F^
Prilled urea	–	2.21^E^	26.5^A^	0.75^D^	20.4^AB^	3.08^C^	9.12^E^
1.0% SCU	2.83	2.50^D^	27.0^A^	0.84^C^	20.8^A^	3.21 (4.22)^BC^	9.61 (5.37)^DE^
2.0% SCU	5.65	2.56^D^	27.3^A^	0.86^BC^	21.1^A^	3.43 (11.4)^B^	10.15 (11.3)^CD^
3.0% SCU	8.48	2.65^C^	27.6^A^	0.89^AB^	21.5^A^	3.68 (19.5)^A^	10.62 (16.5)^BC^
4.0% SCU	11.30	2.75^B^	27.9^A^	0.90^AB^	21.7^A^	3.72 (20.8)^A^	11.16 (22.4)^AB^
5.0% SCU	14.13	2.98^A^	28.2^A^	0.92^A^	21.9^A^	3.85 (25.0)^A^	11.53 (26.4)^A^
Absolute control	–	1.78^D^	23.3^D^	1.46^E^	23.5^B^	3.35^F^	6.13^E^
Prilled urea	–	2.90^BC^	26.5^C^	1.70^D^	25.7^A^	4.76^E^	8.31^D^
0.5% ZnCU (ZnSHH)	1.41	3.20^AB^	27.3^ABC^	1.80^C^	26.1^A^	4.97 (4.41)^DE^	8.63 (3.85)^CD^
0.5% ZnCU (ZnO)	1.41	3.17^AB^	27.1^BC^	1.68^D^	26.0^A^	4.95 (3.99)^DE^	8.58 (3.25)^CD^
1.0% ZnCU (ZnSHH)	2.82	3.26^A^	27.8^ABC^	1.71^D^	26.5^A^	5.23 (9.87)^BCD^	8.93 (7.46)^BC^
1.0% ZnCU (ZnO)	2.82	3.23^AB^	27.6^ABC^	1.70^D^	26.3^A^	5.21 (9.45)^CD^	8.88 (6.86)^BC^
1.5% ZnCU (ZnSHH)	4.23	3.25^A^	28.3^AB^	1.83^C^	26.8^A^	5.41 (13.7)^ABC^	9.25 (11.3)^AB^
1.5% ZnCU (ZnO)	4.23	3.25^A^	28.2^ABC^	1.81^C^	26.6^A^	5.38 (13.1)^ABC^	9.20 (10.7)^AB^
2.0% ZnCU (ZnSHH)	5.64	3.38^A^	28.8^AB^	1.97^B^	27.1^A^	5.56 (16.8)^A^	9.47 (14.0)^A^
2.0% ZnCU (ZnO)	5.64	3.35^A^	28.6^AB^	1.93^B^	27.0^A^	5.52 (16.0)^AB^	9.40 (13.2)^A^
2.5% ZnCU (ZnSHH)	7.05	3.42^A^	29.0^A^	2.10^A^	27.2^A^	5.61 (17.9)^A^	9.59 (15.4)^A^
2.5% ZnCU (ZnO)	7.05	3.49^A^	28.7^AB^	2.05^A^	27.1^A^	5.58 (17.2)^A^	9.51 (14.4)^A^

^a)^ Figures in parenthesis indicate % increase in yield over prilled urea.

Application of 0.3–0.5% BCU significantly increased grain weight panicle^−1^ as compared to 0.1 and 0.2% BCU, PU, and absolute control. Coating of urea with 5.0% S being at par with 3 and 4% SCU and increased grain weight panicle^−1^ as opposed to 1.0 and 2.0% SCU, PU, and absolute control. Application of 2.5% ZnCU (ZnSHH or ZnO) produced the heaviest panicle over other coating materials, PU, and absolute control. BCU, SCU, and ZnCU did not increase 1000 grain weight compared to PU but the weight improved compared to absolute control. Among the BCU treatments, 0.5% BCU recorded the highest grain and straw yield and the figures were superior to 0.1 and 0.2% BCU, PU, and absolute control. SCU (3.0–5.0%) produced significantly more grain and straw yield over PU and absolute control. Coated urea with 2.5% ZnSHH gave the highest grain and straw yield which was similar to that obtained with other ZnCU treatments excluding 0.5 and 1.0% ZnCU, PU, and absolute control (Table 1).

### 2.2 Nitrogen, Sulfur, and Zinc Concentrations in Different Rice Parts

The highest concentration of nitrogen was detected in bran followed by grain, straw, and husk, respectively. Application of 0.2 to 0.5% BCU enhanced the nitrogen concentration in grain, bran, husk, and straw as compared to PU and absolute control (**Table** **2**). On an average, 0.5% BCU increased N concentration in grain by 12% over PU treatment. Similarly, 5.0% SCU significantly improved N concentration of grain, bran, and husk over rest of the treatments with the exception of 4.0% SCU. Nitrogen concentration in straw increased significantly with the application of 1.0 to 5.0% SCU than PU and absolute control. Among ZnCU treatments, the highest N concentration in grain, bran, husk, and straw was recorded with 2.5% ZnCU (ZnSHH) and it was significantly more than the figures obtained with PU, 0.5, 1.0, and 1.5% ZnCU, and absolute control.

**Table 2:** Effect of boron‐coated urea, sulfur‐coated urea, and Zn‐coated urea materials on S, Zn, and N content in different parts of rice grain and straw and N use efficiencies. Means in a column with at least one letter common are not statistically significant using Fisher's least significant difference

Treatment	N content in grain [%]	N content in bran [%]	N content in husk [%]	N content in straw [%]	RE_N_ ^a)^ [%]	AE_N_ ^b)^ [kg grain increased kg^−1^ N applied]	PFP_N_ ^c)^ [kg grain kg^−1^ N applied]
Absolute control	1.19^E^	2.35^D^	0.25^E^	0.88^D^	–	–	–
Prilled urea	1.27^D^	2.51^C^	0.27^E^	0.94^C^	34.83^D^	7.77^D^	25.7^D^
0.1% BCU	1.30^CD^	2.54^C^	0.30^D^	1.01^B^	44.60^CD^	8.67^CD^	26.6^C^
0.2% BCU	1.32^C^	2.63^B^	0.33^C^	1.03^AB^	49.67^BC^	9.20^BCD^	27.1^BC^
0.3% BCU	1.36^B^	2.69^AB^	0.37^B^	1.05^A^	55.87^AB^	9.93^ABC^	27.8^AB^
0.4% BCU	1.39^AB^	2.70^A^	0.39^AB^	1.05^A^	60.20^AB^	10.70^AB^	28.6^AB^
0.5% BCU	1.41^A^	2.73^A^	0.41^A^	1.06^A^	63.20^A^	11.10^A^	29.0^A^
Absolute control	1.19^F^	2.35^D^	0.25^F^	0.88^C^	–	–	–
Prilled urea	1.27^E^	2.51^C^	0.27^EF^	0.94^B^	34.83^E^	7.77^C^	25.7^B^
1.0% SCU	1.29^DE^	2.55^C^	0.29^DE^	1.02^A^	46.87^D^	8.83^BC^	26.8^A^
2.0% SCU	1.32^CD^	2.64^B^	0.31^CD^	1.05^A^	57.20^C^	10.67^B^	28.6^A^
3.0% SCU	1.35^BC^	2.67^B^	0.33^BC^	1.06^A^	66.00^BC^	12.77^A^	30.7^A^
4.0% SCU	1.38^AB^	2.71^AB^	0.35^AB^	1.07^A^	73.00^AB^	13.10^A^	31.0^A^
5.0% SCU	1.39^A^	2.75^A^	0.36^A^	1.07^A^	78.17^A^	14.20^A^	32.1^A^
Absolute control	1.18^H^	2.20^H^	0.54^H^	0.91^G^	–	–	–
Prilled urea	1.29^G^	2.55^G^	0.62^G^	0.95^FG^	34.60^G^	10.83^G^	36.6^E^
0.5% ZnCU (ZnSHH)	1.31^EFG^	2.57^FG^	0.65^EFG^	0.96^EF^	40.50^F^	12.47^F^	38.2^CDE^
0.5% ZnCU (ZnO)	1.30^FG^	2.57^FG^	0.64^FG^	0.95^FG^	38.80^F^	12.30^F^	38.1^DE^
1.0% ZnCU (ZnSHH)	1.34^CDE^	2.62^EF^	0.67^CDEF^	0.99^DEF^	48.60^E^	14.50^DE^	40.2^BC^
1.0% ZnCU (ZnO)	1.32^DEF^	2.61^EFG^	0.65^EFG^	0.97^EF^	45.80^E^	14.30^E^	40.1^BCD^
1.5% ZnCU (ZnSHH)	1.37^BC^	2.65^DE^	0.69^BCD^	1.03^ABCD^	57.00^C^	15.83^BC^	41.6^AB^
1.5% ZnCU (ZnO)	1.35^CD^	2.66^CD^	0.66^DEF^	1.00^CDE^	53.30^D^	15.60^CD^	41.4^AB^
2.0% ZnCU (ZnSHH)	1.40^AB^	2.72^ABC^	0.71^AB^	1.05^AB^	63.00^B^	17.00^AB^	42.8^A^
2.0% ZnCU (ZnO)	1.37^BC^	2.70^BCD^	0.68^BCDE^	1.02^BCD^	58.60^C^	16.70^ABC^	42.5^A^
2.5% ZnCU (ZnSHH)	1.43^A^	2.78^A^	0.73^A^	1.07^A^	67.30^A^	17.37^A^	43.1^A^
2.5% ZnCU (ZnO)	1.41^A^	2.73^AB^	0.70^ABC^	1.04^ABC^	63.30^B^	17.13^A^	42.9^A^

^a)^ RE: Recovery efficiency

^b)^ AE: Agronomic efficiency

^c)^ PFP: Partial factor productivity.

**Figure 1:**
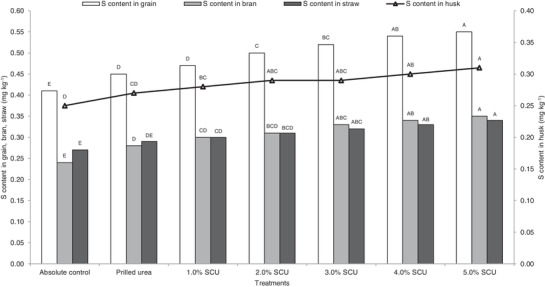
S content in rice grain, bran, husk, and straw as influenced by sulfur‐coated urea. Means for each parameter with at least one letter common are not statistically significant using Fisher's least significant difference.

Application of PU significantly increased the S concentration in grain and bran over absolute control. However, S concentration in husk and straw was similar in PU and absolute control. Coating of urea with 2.0 to 5.0% S significantly improved S concentration in grain over PU and absolute control. Moreover, application of 3.0 to 5.0% SCU increased S concentration in bran, husk, and straw over PU. Among different treatments of SCU, the highest concentration of S in grain, bran, husk, and straw was registered in 5.0% SCU followed by 4.0% SCU (**Figure** **1**).

**Figure 2:**
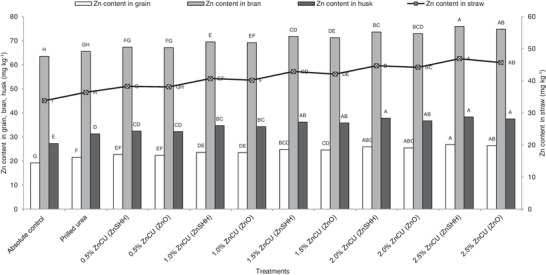
Zn content in rice grain, bran, husk, and straw as influenced by zinc‐coated urea. Means for each parameter with at least one letter common are not statistically significant using Fisher's least significant difference.

Zinc concentration in grain, husk, and straw increased significantly with the application of PU compared to absolute control. Application of 1.0 to 2.5% ZnCU (ZnSHH or ZnO) further improved Zn concentration in grain, bran, husk, and straw over PU treatment. The highest Zn concentration in grain, bran, husk, and straw was registered with the application of 2.5% ZnCU (ZnSHH) closely followed by 2.5% ZnCU (ZnO) and 2.0% ZnCU treatments. Concentration of Zn in grain was 24.7% greater with 2.5% ZnCU (ZnSHH) over PU treatment. Among different sources, ZnSHH‐coated urea was superior to ZnO‐coated urea with respect to improvement in Zn concentration in grain, bran, husk, and straw (**Figure** **2**).

### 2.3 Nitrogen Use Efficiency

The highest agronomic efficiency (AE_N_) was achieved with 0.5% BCU and was significantly superior to PU and BCU (0.1–0.2%). Recovery efficiency (RE_N_) increased from 34.8% (with PU) to 63.2% with 0.5% BCU, while the highest partial factor productivity (PFP_N_) was recorded in 0.5% BCU and it was at par with 0.3–0.4% BCU treatment. With respect to SCU, all treatments recorded similar values of PFP except PU. SCU (4.0–5.0%) recorded significantly higher RE_N_ than PU, 1.0, 2.0, and 3.0% SCU (Table 2).

Among ZnCU treatments, the highest PFP_N_ was registered with 2.5% ZnSHH and was at par with rest of the treatments except 0.5 and 1.0% ZnCU and PU. RE_N_ and AE_N_ also increased with 2.5% ZnSHH, but similar AE_N_ was observed in 2.0% ZnCU. Coating of urea with 2.5% ZnCU almost doubled the recovery of N over PU (Table 2).

### 2.4 Zinc Use Efficiency

The highest RE_Zn_ was found with 1.5% ZnCU along with ZnSHH and it was similar to 1.0 and 2.0% ZnCU (ZnSHH) treatments. RE_N_ increased by 27.4 and 23.9% with 1.5 and 1.0% ZnSHH coated urea, respectively, over 0.5% ZnO coating, while the highest AE_Zn_ was recorded in 1.0% ZnSHH coated urea treatment. The ZnCU and PU treatments did not differ from each other with respect to Zn harvest index (HI_Zn_) but were significantly superior to the absolute control. Among treatments, the highest PFP_Zn_ was registered with 0.5% ZnCU either through ZnSHH or ZnO and it was significantly superior to the rest of the treatments (**Table** **3**).

**Table 3:** Effect of Zn‐coated urea materials on Zn use efficiencies. Means in a column with at least one letter common are not statistically significant using Fisher's least significant difference

Treatment	RE_Zn_ ^a)^ [%]	AE_Zn_ ^b)^ [kg grain increased kg^−1^ Zn applied)	PFP_Zn_ ^c)^ [kg grain kg^−1^ Zn applied]	HI_Zn_ ^d)^ [%]
Absolute control	–	–	–	23.7^B^
Prilled urea	–	–	–	25.3^A^
0.5% ZnCU (ZnSHH)	2.73^C^	148.7^CD^	3524.8^A^	25.4^A^
0.5% ZnCU (ZnO)	2.34^E^	134.7^F^	3510.6^A^	25.3^A^
1.0% ZnCU (ZnSHH)	2.90^AB^	166.1^A^	1848.0^B^	25.3^A^
1.0% ZnCU (ZnO)	2.66^C^	159.0^B^	1841.0^B^	25.5^A^
1.5% ZnCU (ZnSHH)	2.98^A^	153.3^C^	1275.9^C^	25.3^A^
1.5% ZnCU (ZnO)	2.71^C^	146.2^DE^	1268.9^C^	25.5^A^
2.0% ZnCU (ZnSHH)	2.88^AB^	141.6^E^	984.1^D^	25.4^A^
2.0% ZnCU (ZnO)	2.68^C^	134.5^F^	977.0^DE^	25.3^A^
2.5% ZnCU (ZnSHH)	2.77^BC^	120.4^G^	794.6^E^	25.0^A^
2.5% ZnCU (ZnO)	2.51^D^	116.1^G^	790.5^E^	25.3^A^

^a)^ RE: Recovery efficiency

^b)^ AE: Agronomic efficiency

^c)^ PFP: Partial factor productivity

^d)^ HI: Harvest index.

### 2.5 Economics

The cost of inputs for BCU ranged from US$ 4.67 to 23.30 ha^−1^ for 0.1 to 0.5% BCU (**Table** **4**). Total cost for the coating of urea with boron varied from US$ 6.75 ha^−1^ for 0.1% BCU to US$ 26.68 ha^−1^ for 0.5% BCU. Treatment 0.5% BCU was the costliest and was 2.49% higher than PU treatment. Higher gross returns, net returns, and benefit:cost ratio were recorded with the application of 0.3 to 0.5% BCU compared to 0.1 and 0.2% BCU over PU and absolute control. Among BCU treatments, benefit:cost ratio was similar except for 0.1% BCU.

**Table 4:** Cost involved in the coating of boron, sulfur, and zinc onto prilled urea and economic evaluation for one hectare aromatic rice crop. Means in a column with at least one letter common are not statistically significant using Fisher's least significant difference

Treatment	Cost involved					Economic evaluation			
	Quantity [kg ha^−1^] of B/S/Zn required^a)^	Amount of borax/sulfur/Zn product [kg ha^−1^] required^a)^	Cost of borax/sulfur/Zn [US $ ha^−1^]	Cost of coating of urea with B/S/Zn^b)^	Total cost of coating urea of [US $ ha^−1^]	Cultivation cost [US $ ha^−1^]	Gross returns [US $ ha^−1^]	Net returns [US $ ha^−1^]	Benefit:cost ratio
Absolute control	–	–	–	–	–	983.5	1443.8^E^	460.3^E^	0.47^D^
Prilled urea	–	–	–	–	–	1058.6	2057.9^D^	999.4^D^	0.94^C^
0.1% BCU	0.28	2.33	4.67	2.08	6.75	1065.2	2128.2^CD^	1063.0^CD^	1.00^BC^
0.2% BCU	0.56	4.66	9.32	2.40	11.72	1070.2	2170.3^BCD^	1100.1^BCD^	1.03^ABC^
0.3% BCU	0.84	7.00	14.0	2.73	16.73	1075.1	2230.7^ABC^	1155.6^ABC^	1.07^AB^
0.4% BCU	1.12	9.32	18.63	3.05	21.68	1080.0	2291.3^AB^	1211.3^AB^	1.12^AB^
0.5% BCU	1.40	11.65	23.30	3.38	26.68	1085.0	2323.8^A^	1238.8^A^	1.14^A^
Absolute control	–	–	–	–	–	983.5	1443.8^D^	460.3^D^	0.47^D^
Prilled urea	–	–	–	–	–	1058.6	2057.9^C^	999.4^C^	0.94^C^
1.0% SCU	2.83	3.14	1.57	1.85	3.42	1061.9	2144.9^BC^	1082.9^BC^	1.02^BC^
2.0% SCU	5.65	6.28	3.13	1.97	5.10	1063.6	2288.9^B^	1225.2^B^	1.15^B^
3.0% SCU	8.48	9.42	4.72	2.08	6.80	1065.3	2449.0^A^	1383.7^A^	1.30^A^
4.0% SCU	11.30	12.56	6.28	2.18	8.47	1066.9	2486.1^A^	1419.2^A^	1.33^A^
5.0% SCU	14.13	15.70	7.85	2.30	10.15	1068.6	2572.5^A^	1503.9^A^	1.41^A^
Absolute control	–	–	–	–	–	983.5	1533.7^E^	550.2^E^	0.56^F^
Prilled urea	–	–	–	–	–	1058.6	2169.4^D^	1110.8^D^	1.05^E^
0.5% ZnCU (ZnSHH)	1.41	7.07	3.50	1.98	30.20	1064.0	2263.9^CD^	1199.9^CD^	1.13^DE^
0.5% ZnCU (ZnO)	1.41	1.76	2.61	1.91	29.24	1063.1	2254.5^CD^	1191.4^CD^	1.12^DE^
1.0% ZnCU (ZnSHH)	2.82	14.1	6.98	2.23	33.91	1067.8	2378.6^BC^	1310.9^BC^	1.23^BCD^
1.0% ZnCU (ZnO)	2.82	3.52	5.23	2.10	32.05	1065.9	2371.9^BC^	1303.3^BC^	1.22^CD^
1.5% ZnCU (ZnSHH)	4.23	21.15	10.48	2.46	37.66	1071.5	2460.8^AB^	1389.3^AB^	1.30^ABC^
1.5% ZnCU (ZnO)	4.23	5.28	7.84	2.28	34.83	1068.7	2447.2^AB^	1378.5^AB^	1.29^ABC^
2.0% ZnCU (ZnSHH)	5.64	28.2	13.96	2.71	41.39	1075.2	2528.1^A^	1452.9^A^	1.35^AB^
2.0% ZnCU (ZnO)	5.64	7.04	10.46	2.46	37.64	1071.5	2509.9^A^	1438.4^A^	1.34^ABC^
2.5% ZnCU (ZnSHH)	7.05	35.25	17.46	2.95	45.13	1079.0	2551.7^A^	1472.8^A^	1.36^A^
2.5% ZnCU (ZnO)	7.05	8.81	13.09	2.64	40.45	1074.3	2537.4^A^	1463.1^A^	1.36^A^

^a)^ For coating of 282.6 kg urea (kg ha^−1^)

^b)^ At the rate of 7% of fertilizer prices (US $ ha^−1^)

Prevailing prices of fertilizer materials during 2013&14: (i) Borax (12% boron) at the rate of US $ 2.0 kg^−1^, (ii) sulfur dust (90% S) at the rate of US $ 0.50 kg^−1^, (iii) ZnSO_4_·7H_2_O (20% Zn) at the rate of US $ 0.50 kg^−1^; (iv) ZnO (80% Zn) at the rate of US $1.49 kg^−1^, (v) prilled urea at the rate of US $ 0.087 kg^−1^, (vi) price of one US $ = 60.6 INR [Note: Prilled urea at the rate of US $ 883 tonne^−1^; US $ 24.97 for an application of 130 kg ha^−1^].

Total cost incurred for coating urea with S varied from US$ 3.42 ha^−1^ for 1.0% SCU to US$ 10.15 ha^−1^ for 5.0% SCU. Input cost of 1.0 to 0.5% SCU ranged from US$ 1.57 to 7.85 ha^−1^. Gross returns, net returns, and benefit:cost ratio were identical for 3.0 to 5.0% SCU treatments and significantly higher compared to PU, 1.0% SCU, and 2.0% SCU (Table 4).

Coating of urea with ZnO was cheaper over coating with ZnSHH. Total cost involved in coating urea with different concentration of ZnSHH varied from US$ 30.20 to 37.64. However, total cost of urea coating with ZnO varied from US$ 29.24 to 40.45 (0.5 to 2.5% ZnO coated urea). Economics indicated that application of 1.0 to 2.5% ZnCU either ZnSHH or ZnO significantly enhanced gross returns, net returns, and benefit:cost ratio over uncoated PU and 0.5% ZnCU. Higher gross return, net return, and benefit:cost ratio were recorded in ZnSHH‐coated treatment compared to ZnO‐coated treatments.

## 3 Discussion

Application of BCU (0.3–0.5%) contributing 0.84–1.40 kg B ha^−1^ increased rice yield, N uptake, and N use efficiencies (PFP_N_, RE_N_, and AE_N_) as compared to 0.1–0.2% BCU, PU, and control. However, 0.5% BCU had the highest net returns and benefit:cost ratio (Table 4). The recommended range of B is 0.30 to 2 kg B ha^−1^ for B‐deficient Indian soils^20^ and the amount of B supplied by 0.5% BCU (1.40 kg B ha^−1^) was within that range. This experiment also demonstrated that urea application with B enhances N concentration in grain, bran, husk, and straw of rice. It is reported that B and N have a positive interaction that might have helped in increasing N uptake.^19^, ^21^ Since N uptake is directly proportional to RE_N_, an increase in N uptake by rice resulted in corresponding increase in RE_N_. The maximum gross and net returns were obtained with 0.5% BCU, which were 12.9 and 23.9% higher than uncoated PU, respectively. These experimental data substantiate the fact that application of 0.5% BCU is a promising strategy for rice production especially in boron‐deficient soils.

Sulfur fertilization particularly by SCU in cereal–cereal rotations will guarantee consistent availability of S.^18^ A number of researchers have already reported positive response to N and S fertilization in cereals.^1^, ^22^, ^23^, ^24^ There was a significant improvement in PFP_N_, RE_N_, and AE_N_ with SCU as compared to PU (Table 2) as reported earlier by the authors of this manuscript.^1^ Using SCU as source of N and S might have increased N as well as S concentrations, which increased their uptake in grain and straw. Herein, SCU application increased rice yields compared to PU alone (Table 1). This study shows that application of 5% SCU (supplying 14.1 kg S ha^−1^, at application of 130 kg N ha^−1^) enhanced rice productivity, net returns, and benefit:cost ratio. Morris^25^ reported that S recommendation for cereals varies from 10 to 40 kg ha^−1^ and therefore 5% SCU supplied sufficient S to the crop and increased RE_N_ over PU.

The coated urea materials improved Zn content in different rice parts, that is, grain, bran, husk,, and straw which is important for nutritional quality of food and fodder. Results of this study indicate that coated fertilizers with Zn sources significantly improved rice yields as compared to PU (Table 1). Prasad et al.^26^ reported that farmers in African and other developing countries are not adding Zn to soils due to unavailability and higher cost. The yield penalty due to Zn deficiency has been reported in several crops in Asian countries like India, Pakistan, and China, and Australia.^27^ In India, several studies suggested that Zn fertilization increases productivity and profitability of rice and other cereals.^18^, ^28^, ^29^, ^30^, ^31^, ^32^, ^33^, ^34^, ^35^ Among rice parts, Zn concentration decreased in the order bran > straw > husk > grain, indicating that brown rice are much denser in Zn than polished rice grain. Thus, to overcome Zn malnutrition, considering the higher Zn accumulation in the bran, brown rice consumption especially in Asia and Africa could be recommended.^5^ ZnSHH (2.5%) resulted in highest N and Zn uptake in grain, husk, bran, and straw, which was due to increased grain and straw yields, and increased concentration therein. However, Zn coating onto PU differ Zn concentration and their uptake in rice parts between sources with the same level of N input. The mobility of Zn in soil varied among sources which influences Zn concentration and consequently their uptake in different plant parts. In fact, ZnSHH is relatively more water soluble than ZnO in soil which influenced Zn uptake in rice grain parts.^5^, ^18^, ^31^ In this study, ZnCU along with 2.5% ZnSHH led to the highest N concentration in rice grain, husk, bran, and straw which might be due to slow release of N‐coated fertilizers that ultimately increased N uptake.^18^, ^28^, ^36^ Application of 2.5 and 2% ZnSHH resulted in significant increment in RE_N_, PFP_N_, and AE_N_ over PU owing to positive improvement in use efficiencies of N with ZnCU due to more rice yield and N uptake. The highest RE_Zn_ was recorded in 1.5% ZnCU with ZnSHH, while the highest AE_Zn_ and PFP_Zn_ were recorded in 1.0 and 0.5% ZnSHH‐coated urea (Table 3). Zn use efficiencies are high at lower application rates owing to its rapid adsorption over soil organic matter and clay minerals, and subsequent slow desorption.^37^, ^38^ Similarly, Zn‐coated fertilizers would also permit farmers to use Zn along with N in Zn deficient conditions. Among Zn sources, ZnO is easier to coat because it forms a good emulsion with oil.^26^ On the contrary, ZnSHH is a widely used inorganic source of Zn due to its solubility and easier market availability. Overall, coating of urea prills is an option to improve Zn content in rice parts and increase rice yields over PU.

## 4 Conclusion

Coating of urea with different concentrations of B, S, and Zn improves the growth, productivity, and profitability of aromatic rice. BCU, SCU, and ZnCU had beneficial effects in increasing N and Zn concentrations in bran, husk, grain, and straw. Coating of urea with Zn could be used as an effective alternative for ferti‐fortification of Zn in rice grain to reduce Zn deficiency in human beings. Urea coating with 0.5% BCU, 5% SCU, or 2.5% ZnCU resulted in the maximum benefits and increased N as well as Zn use efficiencies.

## 5 Experimental Section

*Description of Study Area*: A field study was carried out during rainy seasons (July–October) of 2013 and 2014 at the Indian Agricultural Research Institute (IARI), New Delhi, India (28°38′N, 77°10′E, 228.6 m above mean sea level). The soil of the experimental site was sandy clay loam (0–20 cm depth) containing 0.49% organic carbon,^39^ 147.3 kg ha^−1^ oxidizable N,^40^ 13.7 kg ha^−1^ available P,^41^ 283.1 kg ha^−1^ exchangeable K,^42^ and pH 8.2 (1:2.5 (soil:water)).^43^ The DTPA‐extractable Zn,^44^ soluble sulfate (estimated turbidimetrically),^45^ and available boron^46^ in the experimental field were 0.56, 10.0, and 0.33 mg kg^−1^ soil, respectively. The critical limits for Zn, B, and S for rice grown in alluvial plains located in rice–wheat belt of north India ranges from 0.38 to 0.90, 0.58, and 8 to 10 mg kg^−1^, respectively.^47^, ^48^

*Experimental Details*: Three coated urea materials, viz., ZnCU, SCU, and BCU were tested in three discreet experiments at the same experimental unit. The experiments were conducted in block design with three replicates. First experiment comprised seven fertilizer treatments, namely, absolute control (no N and no B), PU, and BCU at different proportions (0.1, 0.2, 0.3, 0.4, and 0.5%) while the amount of B applied was 0.28, 0.56, 0.84, 1.12, and 1.40 kg ha^−1^, respectively. In second experiment, instead of BCU, SCU was incorporated at 1, 2, 3, 4, and 5% level and the amount of S applied was 2.83, 5.65, 8.48, 11.3, and 14.13 kg ha^−1^, respectively. Third experiment consisted of 12 combinations of two coating materials, namely, ZnSHH and ZnO with five levels of Zn coating (0.5, 1, 1.5, 2, and 2.5% w/w of PU), PU, and an absolute control (no Zn and no N). The amount of Zn applied was 1.41, 2.82, 4.23, 5.61, and 7.05 kg ha^−1^ with 0.5, 1.0, 1.5, 2.0, and 2.5% ZnCU (ZnSHH or ZnO), respectively. The site was disk‐ploughed thrice and puddled. At final puddling, 26 kg P ha^−1^ as single super phosphate and 33 kg K ha^−1^ as murate of potash were broadcast. Nitrogen at 130 kg ha^−1^ (ZnCU) and 120 kg ha^−1^ (BCU, SCU) as PU or coated fertilizer materials was applied in two equal splits; half at 7 d after transplanting (DAT) and rest half at maximum tillering stage. Rice varieties, viz., Pusa Sugandh 4 (for BCU and SCU experiment) and Pusa Sugandh 5 (for ZnCU experiment), were transplanted with standard agronomic practices in first week of July and harvested in October.

*Prilled Urea Coating Procedure*: Urea‐coated materials with different levels of Zn, S, and B were prepared as per the procedure described by Pooniya et al.^18^ Coated material was prepared just before transplanting of rice. The outlay involved in coating of these urea materials based on prevailing Indian market prices (US $ ha^−1^) during that period is given in Table 4.

*Yield Attributes and Plant Nutrient Analysis*: The rice crop was harvested using sickles as soon as the grain matured after leaving the border area, that is, 0.5 m from all the corners of each plot. Ten panicles from each plot were selected and their length was measured. The crop was threshed using plot thresher. Data were recorded on LAI at 65 DAT, panicle length, grain weight panicle^−1^, 1000 − grain weight, and yields. To calculate grain weight panicle^−1^, ten panicles (selected previously) were threshed and individual grain weight was pooled to determine the average value. For grain yield estimation, moisture content was adjusted at 14% and the straw yield was recorded after sun drying. The recorded yields were expressed in Mg ha^−1^. The input–cost relationships (US $ ha^−1^) for the rice crop is shown in Table 4. The collected plant samples were sundried followed by drying in hot air oven at 65 ± 5 °C, and ground and passed through 40 mesh sieve in a Macro‐Wiley Mill. Samples of 0.5 g dry matter were taken from different parts for N and Zn analysis. Samples were analyzed following Kjeldahl digestion as described by Prasad et al.^43^ Zn content in rice dry matter was determined by a di acid digestion method using atomic absorption spectrophotometry (AAS).^43^ The N or Zn uptake was computed by multiplying their respective concentrations by the mass of rice dry matter. Total N or Zn uptake was calculated by summing up (grain + straw uptake) of N or Zn.

*Nitrogen Use Efficiencies*: Nitrogen use efficiencies, viz., AE_N_, RE_N_, and PFP_N_ were calculated as suggested by Pooniya and Shivay^28^ and Pooniya et al.^18^

(1) $${\mathrm{AE}}_{N}\left(\mathrm{kg}\;\mathrm{grain}\;\mathrm{increased}\;\mathrm{per}\;\mathrm{kg}\;N\;\mathrm{applied}\right)=\left(\mathrm{Yf}-\mathrm{Yc}\right)\mathrm{/Na}$$

(2) $${\mathrm{RE}}_{N}\left(\%\;\mathrm{of}\;N\;\mathrm{taken}\;\mathrm{up}\;\mathrm{by}\;a\;\mathrm{crop}\right)=\left[\left(\mathrm{NUf}-\mathrm{NUc}\right)\mathrm{/Na}\right]\times 100$$

(3) $${\mathrm{PFP}}_{N}\left(\mathrm{kg}\;\mathrm{grain}\;\mathrm{per}\;\mathrm{kg}\;N\;\mathrm{applied}\right)=\mathrm{Yf/Na}$$

where Yf and Yc are the yields (kg ha^−1^) in fertilized and control (no fertilizer) plots, respectively; NUf and NUc are the amounts of N taken up by a rice crop in fertilized and control plots, respectively, and Na refers to the amount of N applied (kg ha^−1^).

*Zinc Use Efficiencies*: Zinc use efficiencies, viz., AE_Zn_, RE_Zn_, PFP_Zn_, and HI_Zn_ were calculated as suggested by Pooniya and Shivay^28^

(4) $${\mathrm{AE}}_{\mathrm{Zn}}\left(\mathrm{kg}\;\mathrm{grain}\;\mathrm{increased}\;\mathrm{per}\;\mathrm{kg}\;\mathrm{Zn}\;\mathrm{applied}\right)=\left(\mathrm{Yf}-\mathrm{Yc}\right)\mathrm{/Zna}$$

(5) $${\mathrm{RE}}_{\mathrm{Zn}}\left(\%\;\mathrm{of}\;\mathrm{Zn}\;\mathrm{taken}\;\mathrm{up}\;\mathrm{by}\;a\;\mathrm{crop}\right)=\left[\left(\mathrm{ZnUf}-\mathrm{ZnUc}\right)\mathrm{/Zna}\right]\times 100$$

(6) $${\mathrm{PFP}}_{\mathrm{Zn}}\left(\mathrm{kg}\;\mathrm{grain}\;\mathrm{per}\;\mathrm{kg}\;\mathrm{Zn}\;\mathrm{applied}\right)=\mathrm{Yf/Zna}$$

(7) $${\mathrm{HI}}_{\mathrm{Zn}}\left(\mathrm{Zinc}\;\mathrm{harvest}\;\mathrm{index}\;\mathrm{as}\%\right)=\left(\mathrm{ZnUg}/\mathrm{ZnUg}+s\right)\times 100$$

where Yf and Yc are the yields (kg ha^−1^) in fertilized and control (no fertilizer) plots, respectively; ZnUf and ZnUc are the amounts of Zn taken up by a rice crop in fertilized and absolute control plots (no N and no Zn), respectively; and Zna refers to the amount of Zn applied (kg ha^−1^). ZnUg and ZnUg + s are the amounts of Zn uptake in rice grain and grain + straw, respectively.

*Statistical Analysis*: The experimental data were investigated statistically using analysis of variance (ANOVA) to determine treatment effects.^49^ Fisher's least significant difference (LSD) was used as a post hoc mean separation test (*P* < 0.05) using Proc GLM in SAS 9.3 software. The Fisher's test was used when the ANOVA was significant.

## Conflict of Interest

The authors declare no conflict of interest.
